# Supplementary figures and images for: Deficiency of malate-aspartate shuttle component SLC25A12 induces pulmonary metastasis
Source: Cancer Metab. 2020 Nov 26;8:26. doi: 10.1186/s40170-020-00232-7 (PMC7690131; doi:10.1186/s40170-020-00232-7)

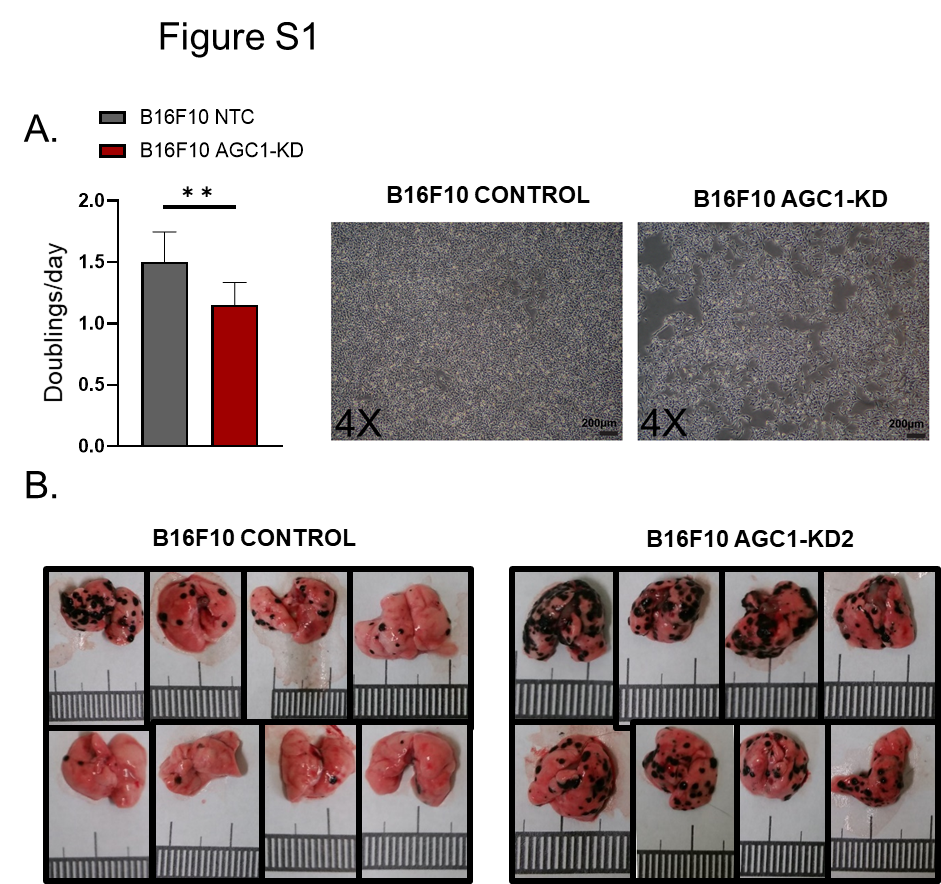

Supplement: Supplementary file 1 — Additional file 1: Figure S1. AGC1-knockdown in B16F10 impairs cell proliferation and increase lung metastasis (A) (left) Proliferation rate of AGC1-KD and control (NTC) B16F10 cells in doublings/day, cultured in DMEM without pyruvate (n = 3) means ±SDs are shown. (right) Representative pictures of B16F10 cells 64 hours after seeding for the proliferation assay. (B) Pictures of lungs with metastasis harvested from mice 16 days after control and AGC1-KD2 (sh908) B16F10 cells were injected intravenously. (related to Fig. 3) [file 40170_2020_232_MOESM1_ESM.docx]

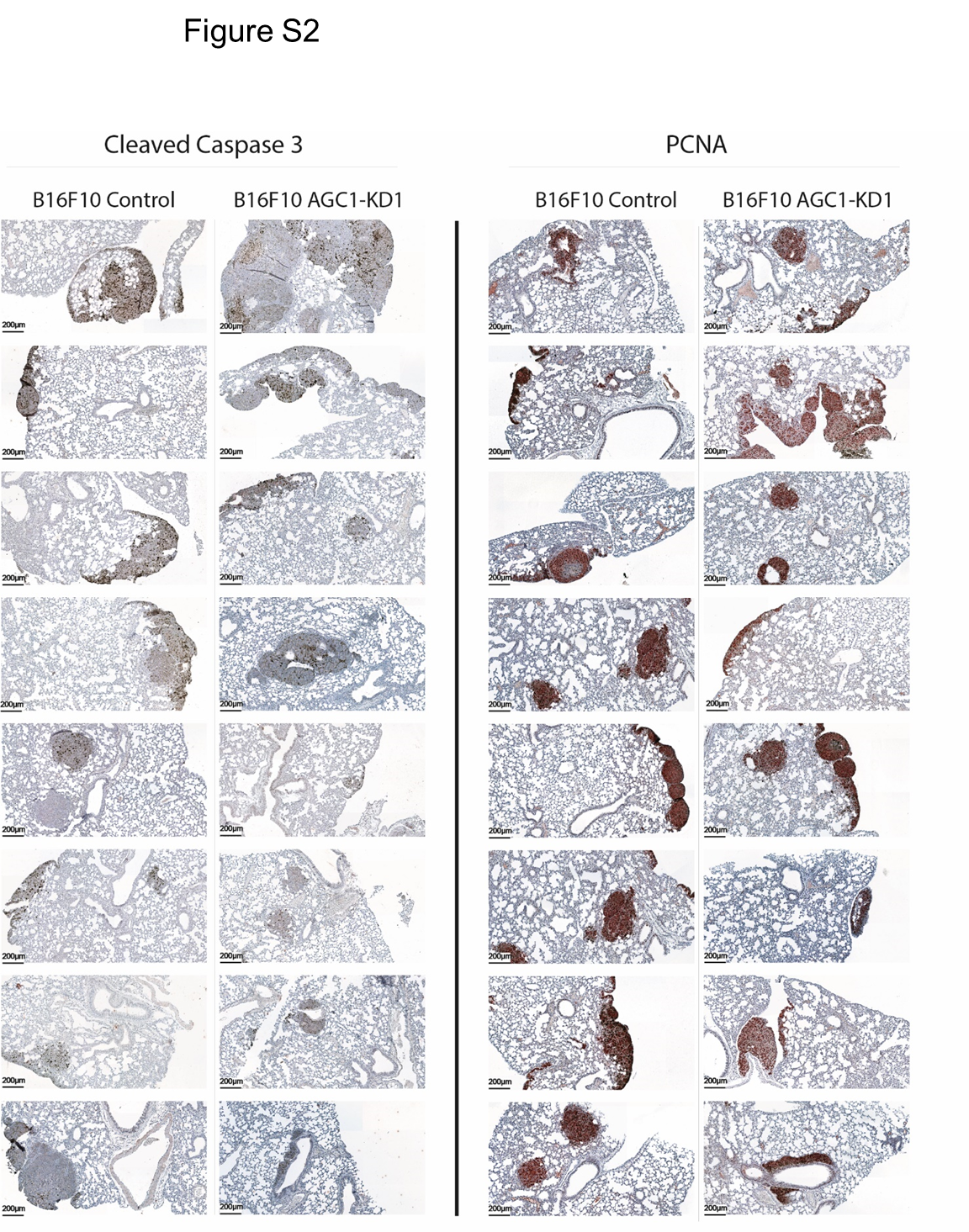

Supplement: Supplementary file 2 — Additional file 2: Figure S2. Expression of apoptosis and proliferation markers are comparable in metastatic AGC1-KD and metastatic control B16F10 tumors. Representative low-magnification images from the IHC stainings of cleaved caspase 3 (CC3) and PCNA of the metastatic regions from B16F10 tumors. Cleaved Caspase 3 protein (CC3) was used as apoptosis marker. PCNA protein were used as proliferation marker. Images were taken at 4X magnification. (related to Fig. 3) [file 40170_2020_232_MOESM2_ESM.docx]

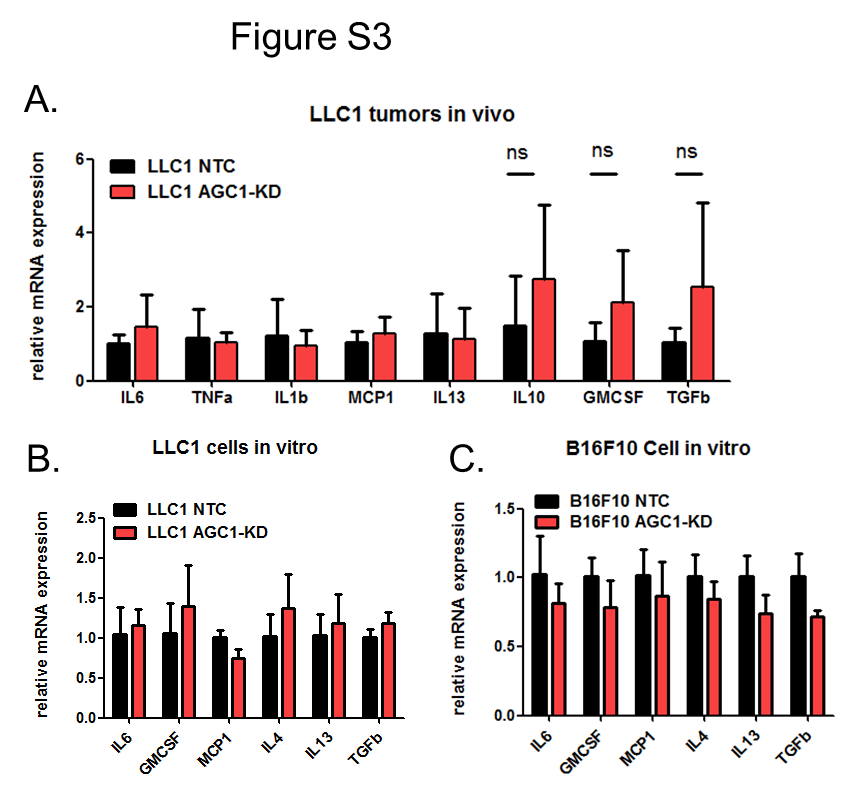

Supplement: Supplementary file 3 — Additional file 3: Figure S3. Levels of metabolites and inflammatory cytokines in AGC1-knockdown tumors (A) Relative mRNA expressions of indicated genes in control (NTC) and AGC1-KD1 LLC1 tumors in vivo (n = 6), means ±SDs are shown. (B-C) Relative mRNA expressions of indicated genes in control (NTC) and AGC1-KD1 (G) LLC1 and (H) B16F10 cells in vitro (n = 3), means ±SDs are shown. Gene names; IL6: Interleukine-6, TNFa:Tumor necrosis factor alpha, IL1b:Interleukine1-beta, MCP1:Monocyte chemoattractant protein-1, IL4: Interleukine-4, IL13:Interleukine-13, IL10:Interleukine-10, GM-CSF :Granulocyte-macrophage colony-stimulating factor, and TGFb:Transforming growth factor beta. (related to Fig. 5) [file 40170_2020_232_MOESM3_ESM.docx]
